# Capsaicin in Metabolic Syndrome

**DOI:** 10.3390/nu10050630

**Published:** 2018-05-17

**Authors:** Sunil K. Panchal, Edward Bliss, Lindsay Brown

**Affiliations:** 1 Functional Foods Research Group, University of Southern Queensland, Toowoomba QLD 4350, Australia; Sunil.Panchal@usq.edu.au (S.K.P.); Edward.Bliss@usq.edu.au (E.B.); 2 School of Health and Wellbeing, University of Southern Queensland, Toowoomba QLD 4350, Australia

**Keywords:** capsaicin, metabolic syndrome, transient receptor potential vanilloid channel 1, TRPV1, obesity, insulin resistance, diabetes, non-alcoholic fatty liver disease

## Abstract

Capsaicin, the major active constituent of chilli, is an agonist on transient receptor potential vanilloid channel 1 (TRPV1). TRPV1 is present on many metabolically active tissues, making it a potentially relevant target for metabolic interventions. Insulin resistance and obesity, being the major components of metabolic syndrome, increase the risk for the development of cardiovascular disease, type 2 diabetes, and non-alcoholic fatty liver disease. In vitro and pre-clinical studies have established the effectiveness of low-dose dietary capsaicin in attenuating metabolic disorders. These responses of capsaicin are mediated through activation of TRPV1, which can then modulate processes such as browning of adipocytes, and activation of metabolic modulators including AMP-activated protein kinase (AMPK), peroxisome proliferator-activated receptor α (PPARα), uncoupling protein 1 (UCP1), and glucagon-like peptide 1 (GLP-1). Modulation of these pathways by capsaicin can increase fat oxidation, improve insulin sensitivity, decrease body fat, and improve heart and liver function. Identifying suitable ways of administering capsaicin at an effective dose would warrant its clinical use through the activation of TRPV1. This review highlights the mechanistic options to improve metabolic syndrome with capsaicin.

## 1. Introduction

### Capsaicin

**Figure 1:**
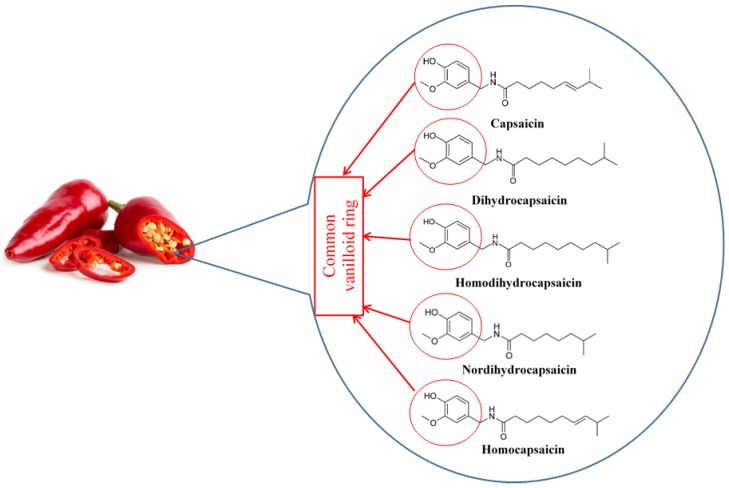
Chemical structure of capsaicinoids.

Capsaicin was first isolated in 1876 [1], its structure was determined in 1919 [2], and it was chemically synthesized in 1930 [3]. Capsaicin, dihydrocapsaicin, nordihydrocapsaicin, homocapsaicin, and homodihydrocapsaicin constitute the capsaicinoids [4] (Figure 1). Capsaicin and dihydrocapsaicin constitute approximately 90% of the capsaicinoids found in any fruit belonging to the Capsicum genus, with capsaicin constituting 70–80% [5,6]. Capsaicin is a pungent molecule that can affect thermoregulation, trigger autonomic reflexes, and is highly absorbed [4,7]. Capsaicin is commercially available as creams and patches for treatment of pain in neuralgias and neuropathies [8,9]. It is in the pipeline for phase III clinical trials as a treatment option for rheumatoid arthritis, postoperative pain, and chronic neuropathic and musculoskeletal pain [7]. Here, we will review the pharmacological potential of capsaicin to reduce metabolic syndrome.

Capsaicin acts through Transient Receptor Potential Channel Vanilloid type-1 (TRPV1) [10,11], a transmembrane cation channel that prefers Ca^2+^ over Na^+^, with six putative transmembrane domains and a calcium-permeable pore region [12,13]. The channel can be activated by many mechanisms, including temperature, low pH, osmotic sensing, taste, pressure, stretch, vibration, and endogenous and exogenous molecules [12,14,15,16,17,18]. TRPV1 acts as the receptor for capsaicin [19], and selective silencing of TRPV1 using specific RNA interference reduced the actions of capsaicin both on calcium influx and inhibition of adipogenesis in 3T3-L1 adipocytes [20]. Although TRPV1 was initially identified on sensory nerve fibers [21], its presence has now been established in many tissues, with highest expression in trigeminal ganglia, dorsal root ganglia, neurons, urinary bladder, and testis [22] with expression in other tissues including adipocytes, smooth muscle cells, endothelial cells, pancreatic β-cells, liver, heart, skeletal muscle, and kidney [23,24,25,26,27,28,29].

Capsaicin binds intracellularly to the vanilloid-binding pocket of TRPV1 and allosterically alters its properties, thus causing an opening of the pore and permitting Ca^2+^ influx [7,30]. This influx of Ca^2+^ causes a change in electrical properties of the cell and allows the release of neurotransmitters, such as substance P and calcitonin gene-related peptide (CGRP) [31,32]. Further, capsaicin increased GLP-1 and decreased ghrelin secretion, indicating a possible interaction between TRPV1 and GLP-1 [33]. TRPV1 was identified on GLP-1-expressing intestinal cells, which upon activation stimulated GLP-1 release [34]. GLP-1 is an incretin hormone that induces expansion of insulin-secreting β-cell mass; this change augments glucose-stimulated insulin secretion [35]. Further, GLP-1 improved insulin sensitivity in humans and rodents [36]. TRPV1 has been identified on pancreatic β-cells, and channel activation has been associated with improved insulin secretion [37]. Clinically, pain management has been the most studied therapeutic response to capsaicin through TRPV1 binding due to the involvement of TRPV1 in pain sensation [28,38,39]. Initially, capsaicin induces pain by sensitization of TRPV1, ultimately leading to inflammation producing pain [28,40]. Exposure to high or repeated doses of capsaicin leads to desensitization of TRPV1, producing analgesia [40]. Apart from pain management, capsaicin may be useful in the treatment of other conditions, such as obesity and cardiovascular disease [38,39]. Further, capsaicin possesses antipruritic, antiinflammatory, antiapoptotic, anticancer, antioxidant, and neuroprotective functions [38]. Capsaicin provides these health benefits through TRPV1 receptors [38].

## 2. Metabolic Disorders

### 2.1. Metabolic Syndrome

Metabolic syndrome is a constellation of symptoms that typically occur together, including central obesity, insulin resistance, hypertension, impaired glucose tolerance, and dyslipidemia [41]. These physiological changes enhance the risk of developing cardiovascular disease, type 2 diabetes, and non-alcoholic fatty liver disease [41,42]. Chronic low-grade inflammation and oxidative stress play important roles in the development of these metabolic complications [43,44,45,46]. In obesity, adipose tissue inflammation increases the production of reactive oxygen species, which then disturbs the production of adipokines, and can trigger pathology associated with metabolic syndrome [46]. Inflammation and oxidative stress are the links between the symptoms of metabolic syndrome, as anti-inflammatory and antioxidant interventions attenuate the changes occurring with metabolic syndrome [47,48]. Obesity and insulin resistance are two major components of metabolic syndrome that predispose individuals to develop further complications of metabolic syndrome. Thus, this review will focus on obesity and insulin resistance as the two major components of metabolic syndrome.

### 2.2. Insulin Resistance and Obesity

Insulin resistance is a reduced response to lowering blood glucose concentrations despite increased insulin concentrations. Many mechanisms have been proposed for the development of insulin resistance, including increased lipid deposition in the tissues [49]. Obesity is an excess deposition of fat in the tissues, especially visceral adipose tissue. Obesity is associated with insulin resistance through chronic low-grade inflammation [50]. Initially, insulin resistance was considered as the major component in the development of metabolic syndrome, and hence, it was called insulin resistance syndrome [51]. This hypothesis was supported by an animal study, which provided evidence that insulin resistance developed before other components of metabolic syndrome [52]. This was also reflected in the definition of metabolic syndrome that was provided by the World Health Organization and European Group for Study of Insulin Resistance [53]. However, the International Diabetes Federation removed the requirement of including insulin resistance as one of the components for diagnosis of metabolic syndrome, instead emphasizing obesity as the defining factor of the syndrome [54]. The incidence of obesity has been escalating throughout the world [55,56]. The increasing incidence of obesity, and therefore, of metabolic syndrome, is primarily due to increasing physical inactivity and the increased acquisition of energy from energy-dense or junk foods [57,58]. Over the years, a clear link has been established between the development of obesity, insulin resistance, and other components of metabolic syndrome [59,60,61,62,63].

### 2.3. Mechanisms for the Development of Insulin Resistance and Obesity

Obesity, dyslipidemia, and reduced physical activity have been identified as major causes of chronic tissue inflammation that contribute to the development of insulin resistance [51]. High-fat diets or overfeeding have been proposed to decrease muscle glucose uptake and increase hepatic gluconeogenesis, both conditions resulting as an outcome of insulin resistance. Insulin resistance in liver and skeletal muscle leads to hyperglycemia, hyperinsulinemia, and then dyslipidemia and fatty liver [64]. Obesity has been linked to the development of cardiac insulin resistance with inflammation, oxidative stress, and dyslipidemia playing an important role in its development [65]. Inflammation in obesity is characterized by increased blood concentrations of inflammatory markers, including tumor necrosis factor (TNF), C-reactive protein, interleukin (IL) 6, and plasminogen activator inhibitor-1 [50]. It has been suggested that inflammation inhibits insulin signaling in adipocytes and hepatocytes through several mechanisms involving inhibition of insulin receptor substrate 1 (IRS-1), insulin receptor, and PPARγ [50]. Although these mechanisms help us to understand how insulin resistance increases the risk for the development of obesity, type 2 diabetes, and cardiovascular disease [50], the exact mechanism for the development of insulin resistance is still a subject of debate [64].

## 3. Capsaicin as a Treatment for Metabolic Syndrome

As mentioned above, TRPV1 is expressed throughout the body in different cells and tissues, including heart, liver, pancreas, kidney, and adipocytes [66]. The presence of TRPV1 on these metabolically active tissues makes capsaicin an important element in targeting metabolic syndrome. Thus, it can be hypothesized that capsaicin plays a crucial role in alleviating symptoms of metabolic syndrome through TRPV1 channels [37,67].

### 3.1. Capsaicin in Insulin Resistance and Glucose Metabolism

Blood glucose regulation is an important homeostatic mechanism for proper cellular functioning [68]. Insulin, as the only hormone that can reduce blood glucose concentrations, plays an important role. Any changes in responses or sensitivity to insulin lead to impairment of glucose tolerance and can result in diabetes.

#### 3.1.1. In Vitro Studies

Capsaicin has been tested in vitro and in vivo for its beneficial effects. Capsaicin increased glucose uptake in C2C12 muscle cells by activating AMPK without affecting insulin signaling molecules such as IRS-1 and Akt [69]. Intestinal glucose absorption was inhibited by capsaicin in vitro when it came in direct contact with mammalian small intestine; the extent of inhibition was dependent on the concentration of capsaicin and the incubation time [70]. Capsaicin pre-treatment increased energy metabolism in human intestinal epithelial cell culture by increasing the expression of glycolytic enzymes triosephosphate isomerase and phosphoglycerate mutase, with increased ATP production in these cells [71]. Capsaicin stimulated GLP-1 secretion from secretin tumor cells in a calcium-dependent manner through TRPV1 activation [34].

#### 3.1.2. Animal Studies

*Capsicum frutescens* extract containing capsaicin reduced blood glucose concentrations and increased plasma insulin concentrations in dogs [72,73]. Capsaicin treatment (0.015% in food for 10 weeks) in mice fed a high-fat diet for 10 weeks lowered obesity, fasting glucose, insulin, leptin, inflammatory markers in adipose tissue and liver, and hepatic triglycerides [74]. Treatment increased adiponectin mRNA/protein in the adipose tissue and PPARα/PGC-1α mRNA in the liver [74]. Thus, improved metabolic and inflammatory status in adipose tissue and liver suggest that dietary capsaicin may reduce insulin resistance [74]. These effects were associated with its dual action on PPARα and TRPV-1 expression/activation [74]. In pancreatectomized diabetic rats, capsaicin and capsiate (nonpungent capsaicin analogue) reduced body weight gain, visceral fat accumulation, and serum leptin, while improving glucose tolerance without modulating energy intake [75]. Some of the responses to capsaicin are highlighted in Table 1.

Both capsaicin and capsiate enhanced first and second phase insulin secretion during hyperglycemic clamp, while only capsiate enhanced hepatic insulin sensitivity during euglycemic hyperinsulinemic clamp [75]. Improved hepatic insulin sensitivity was also associated with reduced hepatic glucose output and increased hepatic glycogen storage. These changes were related to enhanced pAkt/PEPCK and pAMPK signaling pathways [75]. In streptozotocin-induced diabetic rats, capsaicin treatment (6 mg/kg/day) activated TRPV1 in the liver and pancreas [84]. In the liver, capsaicin increased expression of TRPV1, liver X receptor (LXR), and pancreatic duodenal homeobox-1 (PDX-1). LXR and PDX-1 controlled glucose metabolism by regulating expression of glucokinase, GLUT2, phosphoenolpyruvate carboxykinase, and glucose 6-phosphatase. These changes suggest inhibition of gluconeogenesis and activation of glycogen synthesis by capsaicin [84]. In KKAy genetically obese and diabetic mice, capsaicin intervention reduced blood glucose, insulin, and triglyceride concentrations with a reduction in hepatic triglyceride deposition [76]. Furthermore, inflammation in adipose tissue and liver were reduced by capsaicin, with no changes in body weight and adiposity [76]. Hepatic AMPK activation, systemic increases in the concentration of adiponectin, and increased AdipoR2 in liver were suggested as the mechanisms of action of capsaicin in reducing these metabolic complications [76]. In ovariectomized Wistar rats given a 30% sucrose solution for 28 weeks, topical application of capsaicin with exercise ameliorated the symptoms of metabolic syndrome induced by hypoestrogenism by activating AMPK [85].

These effects of capsaicin are suggested to be mediated by the agonist action of capsaicin on TRPV1. However, desensitization of sensory nerves through capsaicin in Zucker rats improved fasting blood glucose concentrations and oral glucose tolerance [86]. These beneficial responses were not accompanied by changes in plasma insulin concentrations, or the liver and muscle contents of glycogen and triglycerides, suggesting no improvement in insulin resistance [86]. On the contrary, insulin secretion was improved following resiniferatoxin (capsaicin analogue) intervention in diabetic rats through desensitization of sensory nerves accompanied by a reduction in plasma dipeptidyl peptidase IV [87]. This response of resiniferatoxin suggests increased GLP-1 and GIP in the blood, and hence, improved insulin secretion and insulin sensitivity. TRPV1-expressing sensory nerve fibers in pancreatic islets play an important role in insulin secretion [88]. Capsaicin administration through injection to Zucker Diabetic Fatty rats prevented increased glucose-induced insulin secretion and improved glucose metabolism. These changes were supported by the loss of TRPV1- and CGRP-co-expressing nerve fibers in the pancreas [88]. Acute capsaicin treatment increased GLP-1 and insulin secretion in wild-type mice in a TRPV1-dependent manner [34]. Chronic dietary capsaicin increased plasma GLP-1 and insulin concentrations, with improved glucose tolerance in wild-type mice; these responses were not seen in TRPV1^−/−^ mice [34]. In *db*/*db* mice, TRPV1 activation by capsaicin improved glucose homeostasis, increased GLP-1 production in distal ileum, and increased plasma GLP-1 concentrations [34].

Capsaicin administration 2 h before exercise in rats increased endurance performance time and plasma concentrations of epinephrine, norepinephrine, glucagon, free fatty acids, and glucose, while decreasing plasma insulin concentrations [89]. Glycogen contents in liver and gastrocnemius muscle in exercising rats treated with capsaicin were higher, suggesting glycogen-sparing effects [89]. In contrast, a capsaicin-supplemented diet in rats did not change glycogen content in the liver and soleus muscle, or the serum glucose, lactate, free fatty acids and glycerol concentrations, at rest and during exercise [90]. However, the weight of epididymal adipose tissue was lower in rats fed a capsaicin-containing diet [90]. Capsaicin was suggested to increase glycogen turnover instead of changing glycogen contents in the liver and soleus muscle in this study [90]. Both capsinoids and capsaicin in mice enhanced energy expenditure and fat oxidation by activating sensory nerves that express TRPV1 in small intestine, and also by enhancing thermogenesis [91]. Capsaicin supplementation in mice improved physical activities, including grip strength and endurance performance, by increasing liver glycogen content [92]. Furthermore, capsaicin decreased exercise-induced fatigue-related parameters, including increased lactate, ammonia, glucose, blood urea nitrogen, and creatine kinase, in a dose-dependent manner [92]. Dihydrocapsaicin decreased body weight gain, and food efficiency, but did not change white and brown adipose tissue (BAT), nor increase plasma concentrations of glucose, free fatty acids, and glycerol [93]. Capsaicin-sensitive sensory nerves were crucial in controlling glucose metabolism and insulin responses following increased glucose load [94,95,96,97,98,99,100]. TRPV1 knockout mice that were fed a high-fat diet became more obese and were more insulin- and leptin-resistant than the wild-type mice fed a high-fat diet [101]. This study indicates that TRPV1 plays an important role played in the development of obesity and insulin resistance associated with high-fat diet and aging. Thus, TRPV1 agonists such as capsaicin may prove highly effective in attenuating metabolic complications [101].

#### 3.1.3. Human Studies

In long-distance male runners (~18–23 years), a single meal with 10g of hot red pepper powder increased respiratory quotient without changes in energy expenditure [102]. Further, results from this study suggested that red pepper ingestion promoted carbohydrate catabolism by increasing plasma epinephrine concentrations [102]. In healthy human subjects, capsaicin increased glucose absorption from the gut, and increased the release of glucagon during glucose loading tests [103]. Intervention with capsaicin-containing chilies for 4 weeks in women with gestational diabetes mellitus reduced postprandial hyperglycemia and hyperinsulinemia, with improved fasting lipid metabolic disorders [82]. These responses further reduced the incidence of large-for-gestational age-newborns. These effects of capsaicin were associated with an increased release of CGRP [82]. In healthy individuals, 5 g capsicum capsules containing 26.6 mg capsaicin lowered plasma glucose concentrations and increased plasma insulin concentrations [104]. In a randomized, double-blind, placebo-controlled, 8 week trial with a combination of nutrients including capsaicin, decreased insulin resistance and inflammatory adipokines were observed, suggesting improved metabolic status [105]. Although studies have identified capsaicin or capsaicinoids responses to insulin secretion or insulin sensitivity in individuals without insulin resistance or hyperinsulinemia [106,107], studies in insulin resistant or hyperinsulinemic individuals would provide suitable evidence for the effects of capsaicin in these individuals.

### 3.2. Capsaicin in Obesity and Dyslipidemia

A meta-analysis of human studies suggested that capsaicin and capsiate can help in weight management [108]. This response of capsaicin has been linked with its capacity to induce thermogenesis and the browning effect in white adipose tissue [109]. Capsaicin administration induced increased UCP1, PPARα, PPARγ, SIRT1, and PRDM-16 expression in adipocytes from mice [110], which indicates browning and thermogenesis.

#### 3.2.1. In Vitro Studies

In 3T3-L1 preadipocytes and adipocytes, low-dose capsaicin lowered PPAR-γ, C/EBP-α, and leptin expression, while inducing apoptosis and anti-adipogenic genes, inhibiting adipogenesis, and promoting brite phenotype in a TRPV1-dependent mechanism [111,112]. At higher doses, capsaicin promoted adipogenesis associated with decreased expression of anti-adipogenic and BAT-specific genes [112]. Capsaicin reduced fatty acid uptake in differentiated Caco-2 cells without activating TRPV1, and increased acetyl-coenzyme A synthetase, suggesting the possibility of inhibiting fatty acid absorption in the intestine with capsaicin [113].

#### 3.2.2. Animal Studies

In high-fat diet-fed rats, capsaicin reduced weight of perirenal adipose tissue and serum triglyceride concentrations [114], and topical capsaicin application reduced body weight and fat gain, as well as reducing fat mass in mesenteric and epididymal adipose tissues [81]. Capsaicin−chitosan microspheres in high-fat diet-fed obese rats reduced obesity more effectively than capsaicin, while decreasing the proportion of body fat. It was proposed that these changes were caused by increases in mRNA and protein expression of PPARα, UCP2, and adiponectin genes, along with downregulating expression of leptin [115]. Capsaicin desensitization of capsaicin-sensitive nerves prevented aging-induced obesity in rats one year after desensitization, indicating a longer-term role for capsaicin in obesity prevention and treatment [116]. Capsaicin in high-fat diet-fed rats decreased serum ALT and AST, along with decreases in serum glucose and HOMA-IR [79]. In high-fat diet-fed mice, combinations of eicosapentaenoic acid and capsaicin reduced body weight, fat tissue weights, serum total cholesterol concentration, and serum activities of AST and ALT, while improving insulin sensitivity [117]. These responses were greater than the response of eicosapentaenoic acid alone in high-fat diet-fed mice [117]. Rats fed high-fat diets were treated with a combination of corn gluten hydrolysate and capsaicin [118]. This combination reduced body weight, fasting plasma glucose, insulin, HOMA-IR, and leptin, while increasing plasma adiponectin [118]. Plasma triglyceride, total cholesterol, HDL-cholesterol, and LDL-cholesterol concentrations were not changed by the combination of corn gluten hydrolysate and capsaicin treatment, whereas hepatic total lipids, triglycerides and total cholesterol concentrations were decreased [118]. It has been suggested that BAT activation, activation of the adreno-sympathetic nervous system for catecholamine production, and increased energy expenditure are responsible for capsaicin’s thermogenic activity [40,109,119]. Capsaicin intervention in high-fat diet-fed rats reduced body weight and adiposity, together with decreases in expression of glyoxalase 1, dihydrolipoamide acetyltransferase, and heat shock protein 27 in skeletal muscle, whereas ATP synthase β subunit, glycogen phosphorylase, and protein phosphatase 1β expression were increased in skeletal muscle [120].

The role of gut microbiota has been well established in metabolic disorders [121,122]. Furthermore, strategies such as prebiotics have been used to successfully modulate gut microbiota, and hence, improve metabolic health [123]. In high-fat diet-fed mice, capsaicin prevented obesity, metabolic endotoxemia, and systemic chronic low-grade inflammation by increasing abundance of butyrate-producing bacteria (*Clostridium* clusters IV (*Ruminococcaceae*) and XIVa (*Lachnospiraceae*, including *Roseburia* spp.)), preventing CB_1_ upregulation, reducing lipopolysaccharides biosynthesis gene expression, and lowering gut permeability [124]. In diabetic *ob*/*ob* mice, capsaicin modulated gut microbiota to improve glucose homeostasis, but failed to reduce obesity [125]. The changes in gut microbiota included increases in *Firmicutes*/*Bacteroidetes* ratio and *Roseburia* abundance, and decreases in the *Bacteroides* and *Parabacteroides* abundances. Furthermore, increases in fecal butyrate and plasma total GLP-1 concentrations, and decreases in plasma concentrations of total ghrelin, TNF, IL-1β, and IL-6, were induced by capsaicin intervention [77]. In high-fat diet-fed mice, capsaicin-induced reduction in body weight and improvements in glucose metabolism were associated with a modulation of gut microbiota, including increases in the proportion of *Bacteroides*, *Coprococcus*, *Prevotella*, and *Akkermansia,* with increases in relative abundance of *Akkermansia muciniphila* [126].

#### 3.2.3. Human Studies

A combination of green tea, capsaicin, and ginger extracts in overweight women decreased body weight, body mass index, serum insulin concentrations, and HOMA-IR, and increased plasma GSH concentrations, but did not affect plasma concentrations of fasting glucose and blood lipid concentrations or plasma total antioxidant capacity [127]. In a randomized, uniform-balanced, crossover design in healthy and active men, a single dose of 1.25 mg capsaicin from cayenne pepper failed to increase thermogenesis and lipid oxidation during rest and exercise [128]. This failure to produce responses by capsaicin may have resulted from the single lower dose of capsaicin used in this study. Thus, a chronic supplementation of capsaicin may have the potential to attenuate obesity.

Modulation of satiety may have a role in capsaicin’s action in obesity. In a controlled feeding trial in healthy subjects, 6 weeks of intervention with capsaicin led to an increased *Firmicutes*/*Bacteroidetes* ratio, abundance of *Faecalibacterium*, plasma GLP-1 and GIP concentrations, and fecal butyrate concentrations, while decreasing plasma ghrelin and lipopolysaccharide binding protein concentrations and fecal Gram-negative bacteria [129]. In this study, these beneficial responses were observed in Bacteroides enterotype subjects, compared to Prevotella enterotype [129]. It was observed that 2.56 mg of capsaicin with every meal in Caucasian subjects increased feelings of satiety and fullness in energy balance, and decreased *ad libitum* intake of food [130]. It was also noted that 1.03 g of red chili pepper containing 2.56 mg capsaicin increased feelings of satiety and fullness, and prevented overeating in Caucasians [130]. In healthy subjects, the consumption of an appetizer with 0.9 g red pepper (0.25% capsaicin) before each meal decreased energy intake over 2 subsequent days [131]. In another study by this group, a red pepper-containing single meal in the postprandial phase did not change feelings of satiety, diet-induced thermogenesis, substrate oxidation, or plasma PYY responses for 3 h. However, this single meal with capsaicin increased plasma GLP-1 concentrations and decreased plasma ghrelin concentrations within 15 min [33]. An intraduodenal infusion of 1.5 mg capsaicin induced satiety in healthy subjects [132]. However, this capsaicin-induced satiety was not associated with changes in plasma concentrations of GLP-1 or PYY, but was associated with general gastrointestinal stress, including pain and nausea [132]. These outcomes suggest that capsaicin may have benefits in inducing satiety. However, most studies were performed in healthy individuals for capsaicin-induced satiety, so clinical measures of changes in obesity are not well understood.

### 3.3. Capsaicin in Vascular and Renal Function

Insulin resistance, type 2 diabetes, and obesity create changes in the vascular environment by increasing glucose, triglycerides, free fatty acids, and LDL-cholesterol, while decreasing HDL-cholesterol in blood [133,134,135,136]. These changes contribute to the development of cardiovascular diseases. Furthermore, components of metabolic syndrome also contribute to changes in kidney function, and may lead to chronic kidney disease [137]. Capsaicin has been studied against these complications, and an association has been identified between dietary capsaicin and a decreased risk for the development of obesity, type 2 diabetes, and cardiovascular diseases [125].

Capsaicin-induced activation of TRPV1 resulted in the release of substance P and CGRP, which produced vasodilation, thus decreasing blood pressure and relieving hypertension [40,138]. Wistar rats with a high intake of salt and who received low-dose capsaicin exhibited increased plasma CGRP concentrations, indicating capsaicin-induced post-synaptic release of CGRP and substance P [138]. When these rats were treated with the TRPV1 antagonist, capsazepine, the blood pressure-lowering effects of capsaicin were ameliorated, thus indicating the direct involvement of endothelial TRPV1 in capsaicin’s response [138]. Chronic low-dose capsaicin treatment reduced blood pressure in Spontaneously Hypertensive Rats, while improving endothelium-dependent relaxation of mesenteric arteries [139]. These rats did not show increased plasma concentrations of CGRP and substance P, suggesting that the long-term consumption of capsaicin reduces arterial pressure, primarily due to the activation of TRPV1, which then promotes phosphorylation of protein kinase A and endothelial nitric oxide synthase. These downstream processes increase the production of nitric oxide, which is responsible for improvements in endothelium-dependent relaxation [139]. Low-dose capsaicin treatment in Stroke-Prone Spontaneously Hypertensive Rats increased eNOS expression in carotid arteries, improved endothelium-dependent relaxation of basilar arteries, decreased intima-media thickness of intracranial arterioles, delayed the onset of stroke, and increased survival time, without changes in blood pressure [140]. TRPV1 activation in renal tissue decreased renal perfusion pressure, increased glomerular filtration rate, and increased water and sodium excretion [141]. This suggests that the TRPV1 activation by capsaicin augments CGRP and substance P release, and therefore, has a key role in mediating renal function and, consequently, blood pressure [141]. Capsaicin reduced plasma total cholesterol and triglycerides concentrations, increased ATP-binding cassette transporter A1 expression, reduced LDL receptor-related protein 1 expression, and reduced lipid storage and atherosclerotic lesions in ApoE^−/−^ mice, but not in the aorta from ApoE^−/−^ TRPV1^−/−^ mice [142]. This study clearly identifies a key role for TRPV1 in the prevention of atherosclerosis [142]. Activation of TRPV1 by capsaicin rescued the autophagy impaired by oxidized LDL by activating the AMPK signaling pathway, and hence, by inhibiting foam cell formation [143]. In *db*/*db* mice, administration of capsaicin reversed high-glucose-induced endothelial dysfunction through TRPV1 activation [144]. It was suggested that this effect may result from increased PKA phosphorylation and upregulation of UCP2 expression. These mechanistic changes reduced oxidative stress and increased NO concentrations [144]. Based on these beneficial responses in a limited number of animal studies, human studies are warranted.

### 3.4. Capsaicin in Non-Alcoholic Fatty Liver Disease

Non-alcoholic fatty liver disease has been considered as one of the consequences of metabolic syndrome [42]. It includes a range of conditions including inflammation, fat deposition, and fibrosis in the liver. Links have been established between obesity, dyslipidemia, and development of non-alcoholic fatty liver disease [145,146]. Dietary capsaicin in high-fat diet-fed mice reduced weight gain and serum activities of ALT and AST, as well as hepatic TNF [147]. Further, hepatic steatosis and inflammation were attenuated by capsaicin, while these effects were absent in TRPV1 knockout mice, suggesting the possible role of TRPV1 in mediating responses of capsaicin [147]. In vitro studies using HepG2 cells confirmed this action to be through PPARδ-mediated enhancement of autophagy [147]. In high-fat diet-fed mice, dietary capsaicin prevented the development of fatty liver, suggesting that UCP2 upregulation is the mechanism [80]. In high-fat diet-fed mice, capsaicin with a combination of antibiotics (vancomycin, neomycin, metronidazole, and ampicillin) reduced intestinal inflammation and leakiness, whereas capsaicin alone was unable to produce these responses. Capsaicin, with or without antibiotics, increased PPARα expression in adipose tissue [148]. Capsaicin and antibiotics had synergistic effects on reducing obesity, fatty liver, and insulin resistance in these high-fat diet-fed mice [148]. Capsaicin attenuated bile duct ligation-induced biliary fibrosis through inhibition of the activation of hepatic stellate cells [149]. Capsaicin also reduced carbon tetrachloride-induced liver fibrosis development in mice [149]. With the limited number of studies of capsaicin in non-alcoholic fatty liver disease in animals, and the absence of studies in humans, this may be an important focus for future research.

### 3.5. Anti-Inflammatory Actions of Capsaicin

A clear link has been established between inflammation and development of metabolic disorders including obesity [150,151,152]. Chronic low-grade inflammation in adipose tissue is now considered an initiator of damage in adipose tissue that contributes to adipogenesis [153]. Thus, anti-inflammatory compounds have been used with success in reducing obesity [154,155,156,157,158,159,160]. Capsaicin has been characterized as an effective anti-inflammatory molecule in metabolic studies [74,76,77,81,105,147]. Based on these outcomes, it can be hypothesized that capsaicin as an effective anti-inflammatory molecule would attenuate metabolic inflammatory conditions including obesity, diabetes, osteoarthritis, and non-alcoholic fatty liver disease.

### 3.6. Capsaicin in Oxidative Stress

Oxidative stress is an important determinant of the development of metabolic syndrome and its associated complications [161,162]. In ovariectomized Wistar rats given 30% sucrose solution, topical application of capsaicin with exercise reduced serum concentrations of malondialdehyde and nitrites, while increasing serum glutathione concentrations and superoxide dismutase activity [85]. In healthy rats, capsaicin reduced oxidative stress, as measured by tissue malondialdehye and diene conjugation [163]. Capsaicin prevented lipid peroxidation and carbonyl formation in proteins in human erythrocytes subjected to oxidative stress [164]. Thus, capsaicin could be effective in reducing the increased oxidative stress that characterizes metabolic disease.

### 3.7. Limitations in Clinical Use of Capsaicin

The strong pungency of capsaicin can limit compliance as a food supplement or nutraceutical, even though capsaicin is commonly consumed through the addition of chilli as a spice in food. This limitation has been overcome in chronic pain management by the use of topical applications, such as creams or patches [8,9]. While this may be feasible in metabolic syndrome, novel products such as chitosan microspheres, liposomes, nanoparticles, or soft gel capsules may be necessary to deliver capsaicin to the intestines, i.e., to bypass release in the stomach [115,165,166]. Furthermore, developing new orally active TRPV1 agonists that mimic the efficacy of capsaicin but lack its pungency could produce an ideal drug for treating metabolic syndrome. An example is the *CH-19* Sweet chilli pepper, containing the three capsaicin analogues, capsiate, dihydrocapsiate, and nordihydrocapsiate [167]. It should be noted that consumption of hot pepper is not equivalent to the use of pure capsaicin [168].

Although capsaicin is commercially available for use in pain management [39,40], adverse effects need to be considered for chronic interventions. Around 10% of patients treated with capsaicin patches reported adverse effects, including erythema and pain at the site of application [39]. Further, capsaicin and capsaicinoids have been shown to induce tumorigenic activities in various cell lines [169]. In contrast, capsaicin reduces the growth of many tumors in mice [169]. Long-term surveillance of chronic capsaicin use in large, multicenter, randomized, double-blinded, and controlled studies in humans is necessary to establish the safety and efficacy of capsaicin and capsaicin analogues.

Functions of mitochondria and endoplasmic reticulum have been identified as playing important roles in skeletal muscle metabolism [170]. Interference with the function of these organelles is an important factor in developing insulin resistance in skeletal muscle [170]. Capsiate has shown beneficial responses in skeletal muscle function [171,172]; thus, capsaicin or its analogues can be targeted for improving skeletal muscle function. Similarly, mitochondrial function in liver was improved by a combination of capsaicin and α-tocopherol [79], further suggesting potential in targeting mitochondrial function with capsaicin.

Although there are appropriate animal studies providing evidence of the potential health benefits of capsaicin in the treatment of metabolic syndrome, the limited number of human studies has not provided the basis for the development of capsaicin as a nutraceutical. Many of the functional foods and nutraceuticals have the same problem, i.e., of not receiving the translation to humans after showing positive effects in animal studies [173]. Furthermore, liver and skeletal muscle play important roles in the metabolism and insulin resistance in these organs; this is an important factor in the development of metabolic syndrome. Thus, identifying responses of capsaicin in humans against insulin resistance in liver and skeletal muscle could provide a valuable foundation for its development as a drug against diabetes and metabolic syndrome. As the adverse effects of capsaicin, such as redness, swelling, soreness, dryness, burning, itching, and pain, are already known through the use of creams and patches, managing similar adverse effects of capsaicin in metabolic syndrome should be carefully considered.

## 4. Conclusions and Future Directions

**Figure 2:**
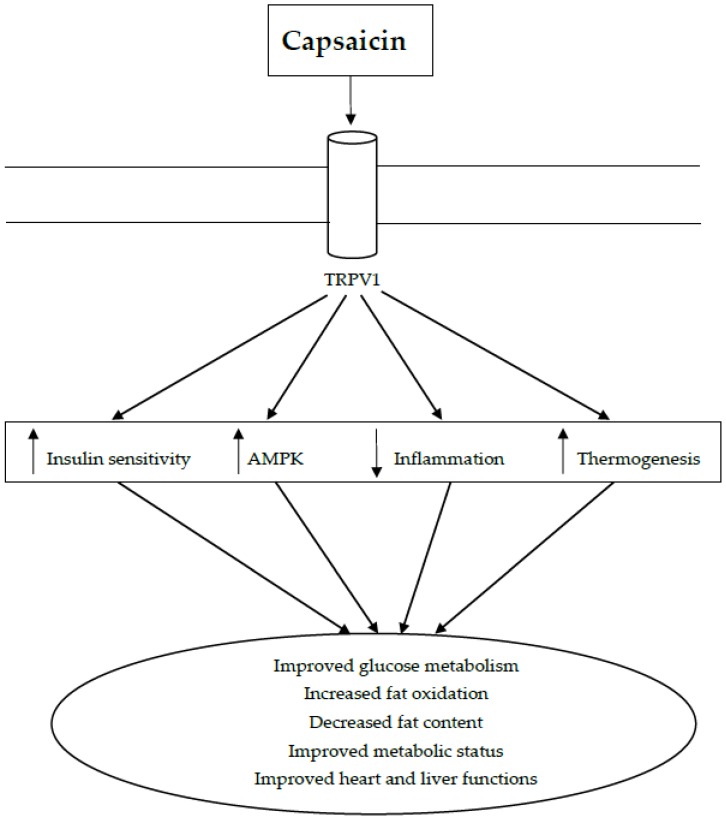
Capsaicin in metabolic syndrome.

Considering the importance of capsaicin-sensitive nerves in controlling glucose metabolism, capsaicin becomes a useful molecule for controlling insulin sensitivity and blood glucose concentrations [98]. Furthermore, the identification of TRPV1 on metabolically-active tissues has also generated interest in the drug design and pharmaceutical industries for targeting TRPV1 to obtain capsaicin-like activity in attenuating metabolic syndrome [37]. A summary of responses to capsaicin through TRVP1 in metabolic disorders is given in Figure 2. Deletion of TRPV1 has further raised the profile of this receptor in treating obesity as the deletion exacerbated diet-induced obesity and insulin resistance [101]. Many agonists for TRPV1 have been tested to identify their potential in improving metabolic complications. Table 2 highlights some of the TRPV1 agonists that were tested against metabolic syndrome.

## Figures and Tables

**Table 1:** A summary of key studies demonstrating the effect of capsaicin on glucose metabolism and insulin responses in animal models and humans.

Animal Model/Human	Capsaicin Dose (Duration)	Effects on Glucose Metabolism	Mechanism(s) to Improve Glucose & Insulin Responses
*db*/*db* mice and TRPV1^−/−^ mice [34]	0.01% of diet (24 weeks)	↑ insulin sensitivity ↓ basal blood glucose	↑ TRPV1 expression ↑ intestinal GLP1 secretion
C57BL/6 mice [74]	0.015% of diet (10 weeks)	↓ basal blood glucose ↓ glucose intolerance ↓ basal blood insulin	↑ TRPV1 activity ↓ PPARγ activity ↓ NF-κB activity ↓ inflammatory cytokines ↓ IRS-1 ↑ GLUT4 expression ↑ adiponectin ↓ leptin
Sprague Dawley rats [75]	0.025% of diet (8 weeks)	↓ basal blood glucose ↓ basal blood insulin ↓ glucose intolerance ↑ insulin sensitivity ↓ insulin intolerance	↓ leptin ↑ pancreatic β-cell mass ↓ pancreatic islet cell apoptosis ↑ ratio of β:α pancreatic cells ↑ pAkt/PEPCK & pAMPK signaling post-TRPV1 activation
KKAy mice [76]	0.015% of diet (3 weeks)	↓ basal blood insulin ↓ basal blood glucose	↓ inflammatory cytokines ↑ adiponectin ↑ AdipoR2 expression
*ob*/*ob* mice [77]	0.01% or 0.02% of diet (6 weeks)	↓ basal blood insulin ↓ basal blood glucose ↓ glucose intolerance ↑ insulin sensitivity ↓ insulin intolerance	↓ ghrelin ↓ inflammatory cytokines ↑ GLP-1 ↑ butyrate
Sprague Dawley rats [78]	Chillies equal to 1% of diet (7 weeks)	↓ basal blood insulin ↓ HOMA-IR ↑ insulin sensitivity ↓ insulin intolerance	↑ pancreatic β-cell mass ↓ pancreatic islet cell apoptosis ↓ β-amyloid accumulation
Swiss albino mice [79]	5 mg/kg/day (8 weeks)	↓ basal blood glucose ↓ HOMA-IR	↑ glucose 6-phosphate dehydrogenase ↑ glutathione-S-transferases
C57BL/6 mice and TRPV1^−/−^ mice [80]	0.01% of diet (24 weeks)	↓ basal blood glucose	↑ hepatic β-oxidation ↑ TRPV1 expression and activity ↑ UCP2 expression and activity
C57BL/6 mice [81]	100 mg of 0.075% capsaicin cream/day (7 weeks)	↓ basal blood glucose ↑ insulin sensitivity	↑ adiponectin ↑ PPARα, PPARγ, visfatin, adipsin ↓ inflammatory cytokines
Women with gestational diabetes mellitus [82]	5 mg/day (4 weeks)	↓ 2 h postprandial blood glucose ↓ 2 h postprandial blood insulin ↓ 2 h postprandial HOMA-IR	↓ calcitonin gene-related peptide
Humans [83]	30 mg/day (4 weeks)	↓ postprandial insulin	↓ postprandial C-peptide ↑ C-peptide/insulin quotient

↑: Increased; ↓: Decreased.

**Table 2:** A summary of studies demonstrating the effects of other TRPV1 agonists on obesity and obesity-related disorders in animal models and humans.

TRPV1 Agonist	Animal Model	Dose (Duration)	Responses
Capsiate [75]	Pancreatectomized rats	0.025% of diet (8 weeks)	↓ body weight gain ↓ visceral fat ↓ leptin ↓ basal blood glucose ↑ glucose tolerance ↑ insulin sensitivity ↑ pancreatic β-cell mass ↓ pancreatic islet cell apoptosis ↑ ratio of β:α pancreatic cells ↓ hepatic triglyceride content ↑ hepatic glycogen content ↑ pAkt/PEPCK & pAMPK signaling post-TRPV1 activation
Dihydrocapsiate [174]	High-fat diet-fed mice	2 mg/kg/day and 10 mg/kg/day (12 weeks)	↓ body weight gain ↓ WAT lipid accumulation ↓ BAT lipid accumulation ↓ hepatic triglyceride content ↓ blood triglycerides ↓ blood insulin ↓ glucose intolerance ↑ energy expenditure & mitochondrial biogenesis gene expression ↑ intestinal crypt depth, muscularis thickness & goblet cells ↓ gut Firmicutes ↓ host energy availability ↑ TRPV1 expression & activity
Resiniferatoxin [112]	High-fat diet-fed mice	200 µg/kg (4 weeks)	↓ body weight gain ↑ locomotor activity
6-gingerol or aza-6-gingerol [175]	High-fat diet-fed mice	0.06% of diet (12 weeks)	↓ body weight gain ↓ visceral fat accumulation ↓ leptin ↓ blood insulin ↓ basal blood glucose ↓ glucose intolerance ↓ hepatic lipogenic enzymes
Piperine [176]	High-carbohydrate, high-fat diet-fed rats	~30 mg/kg/day (8 weeks)	↓ body weight ↓ systolic blood pressure ↓ glucose intolerance ↓ visceral fat accumulation ↓ hepatic fibrosis and fat deposition ↓ cardiac collagen deposition ↑ cardiac function
Nonivamide [177]	Moderately overweight men	0.15 mg/day (12 weeks)	↓ body fat ↑ postprandial serotonin ↑ satiety

↑: Increased; ↓: Decreased.
